# The Outcome of Treatment for Thyroglossal Tract Anomalies

**DOI:** 10.7759/cureus.39325

**Published:** 2023-05-22

**Authors:** Akshaya Rajan, S.M. Azeem Mohiyuddin, Sagayaraj A, Prashanth Babu, Kouser Mohammadi

**Affiliations:** 1 Otorhinolaryngology & Head and Neck Surgery, Sri Devaraj Urs Medical College, Kolar, IND

**Keywords:** thyroglossal tract, recurrence, infrahyoid, thyroglossal cyst, sistrunk

## Abstract

Background

Thyroglossal tract anomalies are the most common cause of midline neck swelling. Thyroglossal cysts present between the base of the tongue and cricoid cartilage as painless, midline swelling that moves on deglutition and protrusion of the tongue. If the thyroglossal cyst gets infected or is violated surgically, it can result in a thyroglossal sinus or fistula. Investigations in patients with suspected thyroglossal cysts include a thyroid function test, ultrasonography of the neck, and fine needle aspiration cytology (FNAC). Computed tomography (CT) or magnetic resonance imaging (MRI) can be done for larger cysts or suspected malignancies. The gold standard treatment is Sistrunk surgery. Recurrence rates with Sistrunk surgery are lower when the surgery is performed accurately. This study was conducted to document the clinical presentation and treatment outcome in patients treated for thyroglossal tract anomalies.

Methods

This is a retrospective analysis of 46 medical case records of patients operated on for thyroglossal tract anomalies at a rural tertiary care hospital from April 1995 to April 2021. Patients fulfilling the inclusion and exclusion criteria were evaluated with a detailed history, various clinical presentations, location, extent of anomalies, and thyroid function test results documented. Ultrasound images were reviewed, and the findings were documented. All patients have consent, and they underwent Sistrunk surgery. Patients in whom the normal thyroid gland was found to be absent were started on replacement thyroxine after surgery. The specimen was subjected to histopathological examination by a senior pathologist. The outcome regarding complications, recurrence, and further treatment were reviewed. The outcome of the thyroglossal fistula was compared with that of thyroglossal cysts, and the outcome of infrahyoid thyroglossal tract anomalies was compared with that of suprahyoid thyroglossal tract anomalies.

Results

In this study, among the 46 patients, 24 (52.2%) were female and 22 (47.8%) were male. The minimum age was three years, the maximum was 58, and the mean was 20.6 years. In this study, 71.7% of the patients were diagnosed with thyroglossal cysts, 10.9% had thyroglossal fistulas, and two had lingual thyroids. The most common location of the cyst was infrahyoid (73.9%). 44 patients underwent Sistrunk surgery, and two patients diagnosed with lingual thyroid underwent excision. Three patients had complications (two pharyngo-cutaneous fistulas, one wound dehiscence), and all were managed conservatively. There were no recurrences in our study.

Conclusion

Thyroglossal tract anomalies are the most common congenital cervical anomalies. A complete Sistrunk procedure includes the removal of the entire thyroglossal tract, inclusive of the body of the hyoid bone along with the cuff of base tongue tissue, and gives the best result for thyroglossal tract anomalies.

## Introduction

Thyroglossal tract anomalies are the most common midline neck swelling, usually between the base of the tongue and cricoid cartilage, and can sometimes arise lower down. The majority of these are sporadic and rarely familial [1].

During the fourth week of gestation, the primitive thyroid develops from the medial anlage and the lateral anlage. The primitive thyroid begins as a ventral diverticulum of endodermal origin between the tuberculum impar and copula in the floor of the pharynx. Later, the tuberculum impar becomes the foramen caecum, and the copula becomes the posterior third of the tongue. As the thyroid descends, it leaves behind an epithelialized tract known as the thyroglossal tract. The developing thyroid descends caudally towards the hyoid bone to reach its final position in the midline of the neck. During maturation, the hyoid bone rotates and draws the thyroglossal tract cranially and posteriorly. The thyroglossal tract persists for six weeks and then atrophies. In the process, the thyroglossal tract can be patent, or thyroid tissue can be left in transit, known as ectopic thyroid. Based on this, the anomaly may only be a cyst or contain ectopic thyroid tissue; if the cyst gets ruptured or infected, it may form a thyroglossal sinus or fistula [1,2].

Thyroglossal cysts are painless, cause midline swelling, and move on to deglutition and protrusion of the tongue. They usually present during childhood or below 20 years of age, but sometimes they can present in middle age [3,4]. 99% of thyroglossal duct cysts are benign, but less than 1% are malignant [4]. Various locations of thyroglossal duct cysts include the infrahyoid, suprahyoid, hyoid, suprasternal, and base of the tongue [1,5]. If the thyroglossal cyst gets infected from an upper respiratory tract infection or is violated surgically, it can result in a thyroglossal sinus or fistula, which presents as painful swelling. In some individuals, thyroid gland remnants grow in the thyroglossal duct or sometimes form a thyroid duct cyst within the thyroid gland (intrathyroidal thyroglossal duct cyst) [6].

Investigations in patients with suspected thyroglossal cysts include a thyroid function test to know the thyroid status of the patient, ultrasonography of the neck to look for calcification, solid components, subclinical lymph nodes, or the presence of a normal thyroid gland, and to guide Fine needle aspiration cytology (FNAC). Computed tomography (CT) or magnetic resonance imaging (MRI) can be done for larger cysts or suspected malignancies. However, thyroglossal duct cysts are commonly confused with other midline neck pathologies. In such scenarios, FNAC plays a pivotal role in establishing the confirmed diagnosis [7].

The gold standard treatment is Sistrunk surgery, which consists of the excision of the cyst, fistula, or sinus along with the tract, a body of the hyoid bone, and a cuff of the base of the tongue tissue (near the foramen caecum); however, suprahyoid cyst surgery does not extend beyond the hyoid bone. Recurrence rates with Sistrunk surgery are lower when the surgery is performed accurately. However, few studies suggest that En bloc removal of the whole central level 1a neck compartment reduces the chance of recurrence significantly [8-10]. In this study, we present our experience with 46 cases of thyroglossal tract anomalies and the outcome of their management.

## Materials and methods

**Figure 1:**
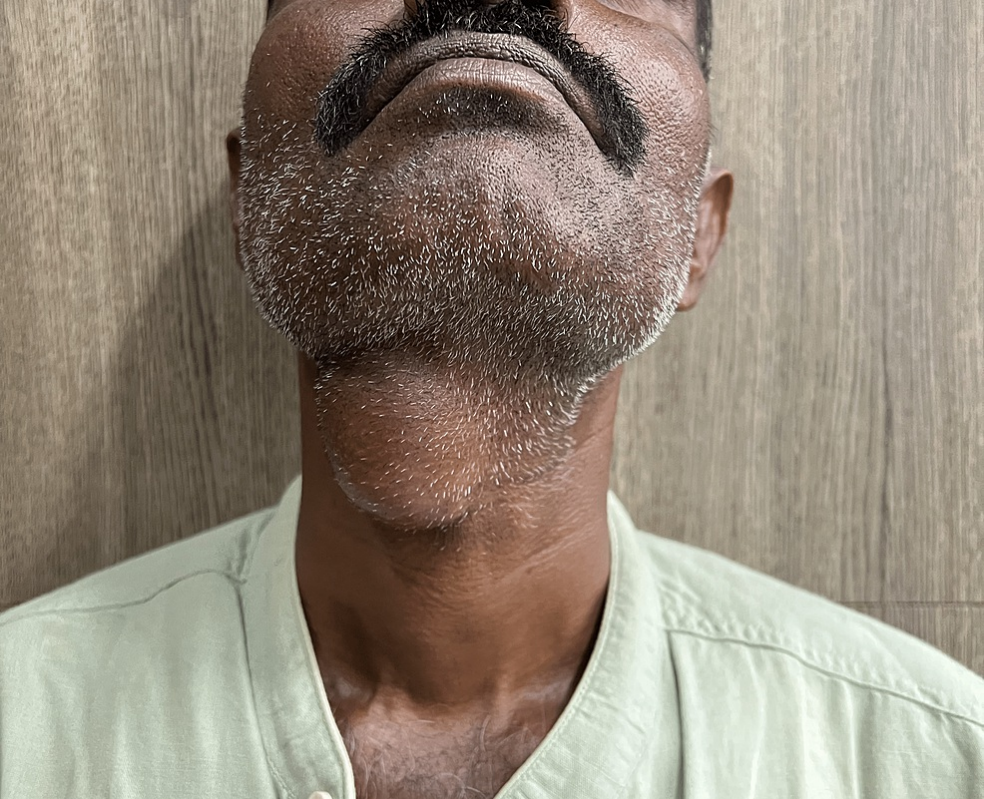
Thyroglossal cyst at the level of the hyoid.

This study was approved by the Institution Ethics Committee (IEC): DMC/KLR/IEC/615/2022-23. This is a retrospective analysis of 46 medical case records of patients operated on for thyroglossal tract anomalies at a rural tertiary care hospital from April 1995 to April 2021. Patients diagnosed as having a thyroglossal cyst, a congenital or acquired thyroglossal fistula or sinus, or a lingual thyroid were included in this study. Patients with midline neck swelling other than thyroglossal tract anomalies like plunging ranula, cystic lymph node, cold abscess, etc., were excluded. Clinical presentations, locations, extent of anomalies, and thyroid function test results were documented. Associated infections and malignancies, if any, were documented. A senior head and neck surgeon did an accurate clinical examination, and the findings were documented (Figure 1).

**Figure 2:**
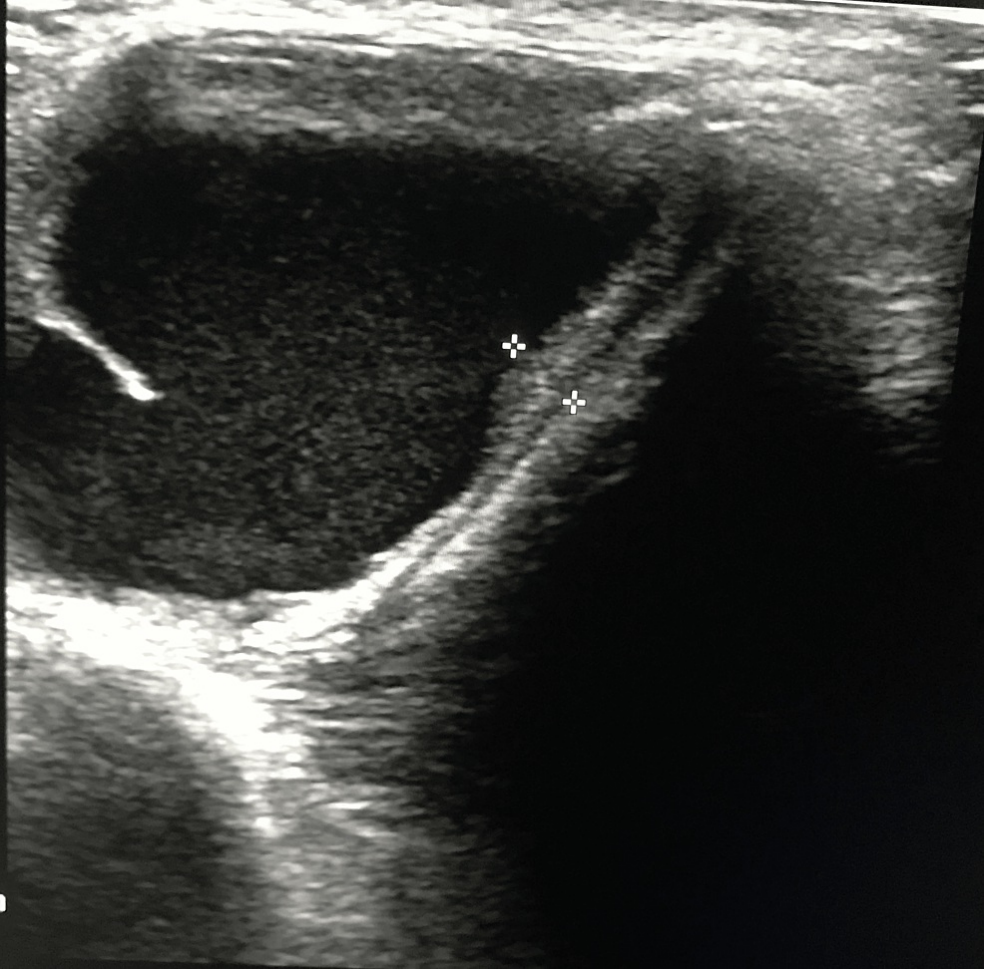
Ultrasonography showing thyroglossal cyst with tract.

Ultrasound imaging was performed to look for the presence of a normal thyroid gland, features of malignancy, subclinical lymph nodes, if any, and their relationship to normal structures. Findings were documented (Figure 2).

After obtaining consent, all patients underwent Sistrunk surgery. Patients in whom the normal thyroid gland was found to be absent were started on replacement thyroxine after surgery. The specimen was subjected to histopathological examination by a senior pathologist. The outcome in terms of complications, recurrence, and further treatment was documented. The outcome of the thyroglossal fistula was compared with the outcome of thyroglossal cysts, and the outcome of infrahyoid thyroglossal tract anomalies was compared with that of suprahyoid thyroglossal tract anomalies. The mean follow-up period was 18 months. Findings were documented in a Statistical Package for Social Sciences (SPSS) Excel sheet, and descriptive results were analyzed.

## Results

In this study, among the 46 patients, 24 (52.2%) were female, and 22 (47.8%) were male. There were 20 pediatric patients (below the age of 15 years). The minimum age was three years, the maximum was 58 years, and the mean was 20.6 years. The distribution of patients according to diagnosis is depicted in Table 1.

**Table 1:** Distribution of patients according to diagnosis

Diagnosis	Frequency	Percent
Thyroglossal cyst	33	71.7
Thyroglossal fistula	5	10.9
Infected thyroglossal cyst with sinus abscess	1	2.2
Lingual thyroid	2	4.3
Thyroglossal cyst with ectopic thyroid	3	6.5
Recurrent thyroglossal cyst	1	2.2
Infected thyroglossal fistula communicating with the pharynx and extending till the base of the tongue	1	2.2
Total	46	100.0

In this study, most of the patients were diagnosed with thyroglossal cysts: 33 (71.7%), among them, 11 were in the pediatric age group, five patients (10.9%) had a thyroglossal fistula (four pediatric and one adult), two patients had lingual thyroid (one pediatric and one adult), and patients with other varied diagnoses included three patients with ectopic thyroids (two pediatric and one adult), among which one had a follicular adenoma. Two patients had infected thyroglossal cysts, among which one patient had a thyroglossal fistula communicating with the pharynx and extending to the base of the tongue. One pediatric patient had an infected thyroglossal cyst with a sinus, and one had a recurrent thyroglossal cyst. The patients' distribution according to the cysts' location is depicted in Table 2.

**Table 2:** Distribution of patients according to the location of cysts

Location	Frequency	Percent
Infrahyoid	34	73.9
Suprahyoid	5	10.9
Hyoid	3	6.5
Lingual	2	4.3
Pre-Tracheal	1	2.2
Suprasternal	1	2.2
Total	46	100.0

Among the 46 patients, two had thyroglossal cysts at the level of the hyoid, and one pediatric patient had a thyroglossal fistula at the same location. Thirty-four patients had infrahyoid thyroglossal tract anomalies, among which 26 patients had thyroglossal cysts (eight pediatric and 18 adults); four patients had thyroglossal fistulas (three pediatric and one adult); one thyroglossal fistula communicating with the pharynx and extending till the base of the tongue; one pediatric patient had infected thyroglossal cyst with a sinus; one thyroglossal cyst with an ectopic thyroid; and one pediatric patient with a recurrent thyroglossal cyst. Two patients had lingual thyroids (one pediatric and one adult). One pediatric patient had a pre-tracheal thyroglossal cyst containing an ectopic thyroid. Five patients had suprahyoid thyroglossal cysts (three pediatric and two adults), and one pediatric patient had ectopic thyroid tissue in the cyst. One pediatric patient had a suprasternal thyroglossal cyst. The distribution of patients according to treatment is depicted in Table 3.

**Table 3:** Distribution of patients according to treatment

Surgery	Frequency	Percent
SISTRUNK	44	95.7
Midline glossotomy with excision	1	2.2
Trans-oral excision	1	2.2
Total	46	100.0

In this study, 44 patients underwent Sistrunk surgery (ten pediatric and 34 adults), and among the two patients diagnosed with lingual thyroid, one patient underwent a midline glossotomy with excision, and one pediatric patient underwent a trans-oral excision. Among the 46 patients, 43 patients, including the two lingual thyroids, had normal thyroid glands, and in three patients (two pediatric and one adult), the thyroid gland was absent. In one pediatric patient, the body of the hyoid was partially eroded due to pressure. The three patients with absent thyroids were later started on replacement thyroxine as they were in a hypothyroid state; other patients had normal thyroid function. On comparing postoperative complications and presentation of cysts, two pediatric and one adult patient had complications (two pharyngo-cutaneous fistulas, one wound dehiscence), and all had an infrahyoid presentation. One pediatric patient with a thyroglossal cyst and one adult patient with a thyroglossal fistula communicating with the pharynx and extending to the base of the tongue developed a pharyngo-cutaneous fistula, which healed spontaneously. One pediatric patient with an infected thyroglossal cyst with sinus had wound dehiscence managed conservatively. There were no recurrences in our study.

## Discussion

During embryonic life, the medial and lateral anlage fuse. During descent, it is closely related to the hyoid primordium and can be anterior, posterior, or around. It often arborizes and has branches around the arch of the hyoid bone. The remnant tract may not obliterate in 7% of the population, resulting in a patent thyroglossal duct, a partially fibrosed tract, or a thyroglossal cyst. It may also contain ectopic thyroid tissue [11-13]. 

In our study, there was no gender predisposition with a mean age of 20.6 years, similar to the study reported by Ubayasiri et al. [12] but different from the series reported by Patigaroo et al. [13], where most of the patients were males with a mean age of 10 years.

In our study, 71.7% of patients presented with painless neck swelling; they were diagnosed as thyroglossal cysts, similar to a study done by Vikas Sinha et al. [14], where 72% presented with painless thyroglossal cysts. In our study, 4.3% of patients presented with an infected thyroglossal cyst; this was in contradiction to the literature, where studies done by Daan Rohof et al. [15] and Zafer et al. [3] showed infection rates of 43% and 33%, respectively.

 The most common location of cysts was infrahyoid (73.9%), similar to Patigaroo et al. [13] and Daan Rohof et al. [15]studies with 83% and 67%, respectively. Although infrahyoid is the most common location, we had other varied locations in our study, like 4.3% having lingual, 2.2% having pre-tracheal, and 2.2% having suprasternal. Three (6.5%) of our patients had only ectopic thyroid tissue without a thyroid gland.

 In our series, 95.6% (44 patients) underwent Sistrunk surgery, and two patients with lingual thyroids underwent excision. Three patients (6.5%) had minor complications (two pharyngo-cutaneous fistulae and one wound dehiscence); they were managed conservatively. There were no recurrences in our study. The low complication rates and absence of recurrence in our series can be explained by the fact that all patients except lingual thyroid underwent the complete Sistrunk procedure (complete removal of the thyroglossal tract with a body of hyoid bone along with cuff of base tongue removal) and all surgeries were performed by a senior surgeon, unlike the Ubayashree et al. series [12], where registrar-junior surgeons operated on 45% of patients with a recurrence rate of 6% and a postoperative complications rate of 5%. Another study done by Daan Rohof et al. [15] showed a 55.6% recurrence rate after simple excision of the thyroglossal tract and a 5.3% recurrence rate after Sistrunk surgery. The low complication rates in our series can also be attributed to the fact that only 4.3% (two patients) presented with infected cysts, unlike in a study done by Daan Rohof et al. [15], where 43% of patients presented with infectious cysts with 5.3% recurrence after Sistrunk surgery, 3.4% major complications, and 11% minor complications. As per the literature, the chance of malignancy is less than 1% [4], whereas in our series, one patient with ectopic thyroid tissue had a follicular adenoma, and there were no malignancies.

On comparing pediatric and adult patients, the occurrence of thyroglossal fistulas and infected sinus was higher in the pediatric age group; this could be attributed to the fact that the pediatric population is more prone to upper respiratory tract infection, which is a predisposing factor as per literature [6]. Further non-compliance by pediatric patients by extending the neck and touching the wound with unsterile hands can also be a risk factor for postoperative wound complications.

Sistrunk surgery, therefore, remains the procedure of choice for thyroglossal tract anomalies, minimizing complications and recurrences. Infected thyroglossal cysts or fistulas can have higher complication rates. Though the procedure was explained in the 1920s, classical Sistrunk surgery is still the best in preventing recurrences. When it is difficult to follow the thyroglossal tract above the hyoid bone, a midline cuff of tissue can be taken in continuity with the body of the hyoid bone, along with a cuff of tissue near the base of the tongue. A meticulous two-layered closure eliminates the chances of wound breakdown or leakage.

Limitations

This is a retrospective study with small sample size, and the follow-up period could have been more.

## Conclusions

Thyroglossal tract anomalies are the most common congenital cervical anomalies. The majority of them are present in childhood or early teenage years, with no gender predisposition. The most common location of a thyroglossal cyst is the infrahyoid. A complete Sistrunk procedure includes the removal of the entire thyroglossal tract, including the body of the hyoid bone, along with the cuff of base tongue tissue. It gives the best result for thyroglossal tract anomalies. Infected thyroglossal cysts, sinuses, or fistulae are more predisposed to complications after surgery. A small percentage of thyroglossal tract anomalies can have only ectopic thyroid tissue without the thyroid gland.
